# A tongue ulcer

**DOI:** 10.11604/pamj.2018.30.31.15007

**Published:** 2018-05-16

**Authors:** Aryé Weinberg, Ralf Siegert

**Affiliations:** 1 Prosper-Hospital, Department of Otorhinolaryngology, Head and Neck Surgery, Recklinghausen, Germany

**Keywords:** Tongue ulcer, pressure ulcer, sharp tooth surface

## Image in medicine

An 86-year-old man presented with a painful lesion on his left tongue measuring 0.6 x 0.6mm (A). The lesion has been growing over the last 2 weeks. The patient did not remember any trauma except that his teeth did sometimes scratch his tongue. Histology form the excised lesion revealed a pressure ulcer (B). Tongue ulcers can be caused by accidental bites, ill-fitting dentures, braces, citrus or acidic fruits and by a sharp tooth surface. In some other cases nutritional problems such as vitamin B12, iron or folic acid deficiency may cause tongue ulcers. Never the less malignancy needs to be excluded. Therefore, complete excision and diagnosis based on histology is recommended.

**Figure 1:**
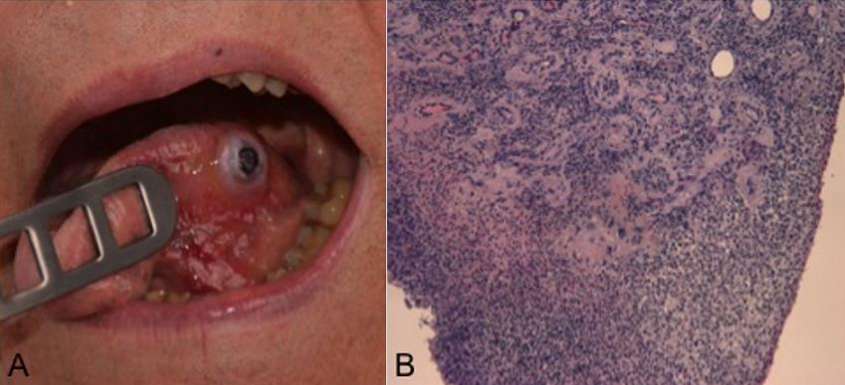
A) tongue lesion; B) pressure ulcer

